# Dynamic life cycle impact assessment (DLCIA) in a sustainable building planning process

**DOI:** 10.1038/s41598-025-20599-1

**Published:** 2025-09-23

**Authors:** Chujun Zong, Farzan Banihashemi, Werner Lang

**Affiliations:** Chair of Energy Efficient and Sustainable Design and Building, Technical University of Munich, Munich, Germany

**Keywords:** Environmental impact, Civil engineering

## Abstract

Building sector significantly contributes to the anthropogenic environmental impacts. To promote sustainable construction, life cycle assessment (LCA) serves as a useful tool to quantify the environmental impacts of a building and suggest improvement solutions. It is widely acknowledged by various regional and international regulations and is mandated for new buildings in the European Union. While the conventional LCA often fails to capture the dynamics in a building’s life cycle, this study enhances the plausibility of building LCA by implementing a dynamic life cycle impact assessment (DLCIA) for a solid masonry building, evaluating improvement solutions across its product and End-of-Life (EoL) phases. Our DLCIA approach, aligned with Intergovernmental Panel on Climate Change Assessment Report (IPCC AR) 6, provides a transparent and clear calculation process of global warming potential (GWP) overtime and a comprehensible comparison with the conventional static calculation. We demonstrate that the dynamic GWP calculations based on the DLCIA approach are consistently 5–7% higher than static counterparts, successfully capturing the continuous decay of GHG emissions in the atmosphere and the environmental impact of each emission event. Applied to improvement solutions, this method guided strategies that achieved a 31.52% reduction in total GWP. Furthermore, integrating a dynamic circular recycling model into the improvement solutions in the EoL phases highlights the potential of combining multiple dynamic factors and critically discusses the concept of circular economy. To promote circular economy in construction, it is essential not only to improve the conversion rate of waste materials into compounds for virgin materials but also to explore cross-material recycling and recycling among multiple building entities. Future dynamic LCA (DLCA) studies should integrate DLCIA with other dynamic factors to enable more comprehensive and systematic assessments.

## Introduction

Globally, the building sector accounts for about 21% of the total greenhouse gas (GHG) emissions^1^ and the whole life cycle of material production, building construction, demolition and recycling is one of the most important supply chains nowadays. It thus emphasizes the importance of quantifying the environmental impacts of buildings to find improvement solutions and realize sustainable construction. For this purpose, life cycle assessment (LCA) serves as a common instrument to quantify the environmental impacts throughout the whole life cycle of a building and is increasingly used in the planning phase to guide decision-making processes. It is widely acknowledged by various regional and international regulations, such as EN 15978 and EN 14040, and is mandated for new buildings in the European Union according the Energy Performance of Buildings Directive. However, the current LCA approaches in practice, especially in the building sector, fail to model the temporal dynamics in the life cycle. Lacking dynamic factors not only weakens the plausibility of the LCA results but also influences the decisions regarding sustainability. To deal with this problem, many studies were carried out in the last decade to discuss possible dynamic LCA (DLCA) methodologies.

The concept of DLCA appears in building industry in 2013, when Collinge et al.^2^ introduced the concept of DLCA in the building industry, categorizing the dynamics in LCA into dynamic life cycle inventory (DLCI) and dynamic life cycle impact assessment (DLCIA). Following this work, numerous studies investigated the concrete factors affecting the LCA results from a temporal perspective. These dynamic factors can be categorized into two groups^2–5^: background dynamic factors and foreground dynamic factors. The latter category refers to the dynamic factors that occur in the foreground data relevant to specific field. Particularly in the building sector, the most studied foreground dynamic factor is the dynamic end use energy consumption^2,6–17^, followed by the dynamic performance of heating, ventilation and air conditioning (HVAC) systems^6,12,14,18,19^, dynamic building areas subject to the population change^12,20^ and dynamic occupant behavior^14^. While the foreground dynamic factors can be modeled by LCA practitioners using the foreground database with the unit values of environmental impacts, background dynamic factors occur in the background database and can be difficult for LCA practitioners to model. Examples of the background dynamic factors include electricity mix^2,7,12,14,17–19,21–26^, material flow after end-of-life^2,10,12,14,18,27–30^, technological improvement^12,14,19,31,32^, domestic production and import or environmental impact calculation, forest growth^23,33^.

Compared to the foreground dynamic factors, background dynamic factors fundamentally influence the unit values of the environmental impact and are the essential part of understanding DLCA conceptually. And among all background dynamic factors, the dynamic environmental impact calculation, i.e., DLCIA is gaining increasing attention. Not only does it build up the dynamics in ecological systems over time instead of applying a cumulative value, but it also enhances the understanding of the original concept of the various environmental impacts, with GWP being the most studied one. However, despite the importance of DLCIA and several promising previous studies, there are certain main problems of DLCIA in the building industry. First, the concept of DLCIA remains abstract. The use of “dynamic characterization factor” as a direct synonym introduces conceptual confusion, as the former is a specific metric within the broader DLCIA framework. Due to the nontransparent original calculation logic of the environmental impacts, e.g., GWP, researchers tried to model the DLCIA by developing proxies based on presumed mathematical relations. For example, Mosquini et al.^34^ applied the linear decreasing weighting factor to represent the DLCIA calculation. Similarly, Andersen et al.^32^ and Resch et al.^31^ used exponential discounting factor. Although applying linear decreasing weighting factor or exponential discounting factor provides a simplified way to break down the cumulative GWP to temporal values, the calculation is not accurate, given the assumption of a simple linear or exponential characteristic. In contrast, other studies performed the DLCIA calculation with the direct calculation of radiative forcing, following the original calculation logic of GWP^2,10,12,23,25,35–42^. Among these existing studies, the works of Zieger et al.^40^ and Cordier et al.^38^ explicitly presented the DLCIA calculation process by providing transparent mathematical equations for the absolute GWP (AGWP). They contributed to render the DLCIA methodologies comprehensible and reproducible. However, the two studies applied the calculation methodology from IPCC Assessment Report 5^43^ or earlier versions that does not consider the carbon response of other gases besides CO$_2$ and an updated calculation is required. In addition, in their comparison of dynamic and static results, they fail to effectively link the DLCIA output to a time-dependent characterization factor for calculating the relative GWP. This limitation obscures the significance and comparative advantage of the DLCIA. Therefore, a transparent and comprehensible presentation of conducting DLCIA and a effective comparative analysis with static methods are important, such that the aforementioned issues can be resolved and the importance of integrating DLCIA into the building planning process can be demonstrated.

In this study, we perform a DLCIA calculation of a case study building to assess its dynamic environmental impact in the form of GWP throughout the whole life cycle and highlight the importance of using DLCIA in the building planing process. Specifically, a solid masonry construction typology is studied and different improvement solutions focusing on construction materials to achieve a sustainable construction are assessed. This work has the following contributions:

- Transparent and up-to-date calculation of DLCIA including the carbon response of all GHG gases.
- Clear and transparent comparison of the static and dynamic results.
- Discussion of possible improvement solutions for building materials in the product and End-of-Life (EoL) phases according to the DLCIA results and analyses.
- Inclusion of dynamic modeling of material flows in the improvement solutions in the EoL phases to combine DLCIA with other dynamic factors.
- Highlight of the importance of DLCIA in the modeling of environmental impact of a building.

## Methods

**Fig. 1:**
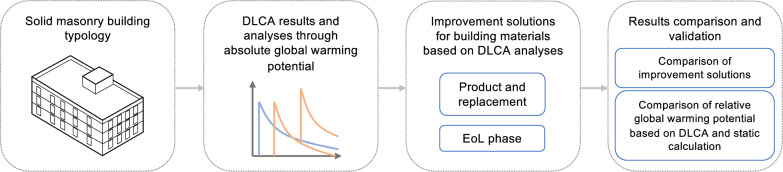
Methodological workflow.

Figure 1 shows an overview of the methodological workflow of this study. We apply a DLCA approach to a solid masonry building typology using the dynamic environmental impact calculation, i.e., DLCIA. The results are analyzed from a temporal perspective to better understand the material characteristics. Subsequently, improvement solutions in both the product and End-of-Life (EoL) phases of building materials are modeled and evaluated using DLCIA to examine their effectivity of reducing the environmental impact. Herein, the improvement solutions in both the product and EoL phases also reflect the material replacements during the building operation. These improvement solutions are analyzed both individually and in combination to provide recommendations for achieving more sustainable building construction practices.

### Data collection

In this study, we identify the materials in different building components of the solid masonry typology, based on the new construction building component catalog from a previous study^44^.The investigated building components include: base plate (BP), floor (FL), exterior wall (EW), flat roof (FRO) and window (WIN). For each building component, the materials are mapped with the activities from ecoinvent 3.11 (see supplementary material). Due to the data transparency and to enable the consistency of modeling, the study is conducted with data of the geographical context of Switzerland. The LCI values are then extracted from ecoinvent for use in the DLCIA calculation. As for the mass of each material, it is determined based on its properties, i.e., thickness, density, thermal conductivity, and the area of building components as derived from a case study building.

The investigated building is a campus facility constructed in 1968 on the main campus of the Technical University of Munich. In line with the nationwide non-residential building research project EnOB:dataNWG^45^, it is classified as a “Building for research and university teaching.” Based on the research in^46^, the original building age class was assigned to Group 1, constructed before 1918. Table 1 summarizes the original thermal transmittance (U-values) and values proposed for a new construction scenario, where the case study maintains the original geometry but adopts the modern standard.

**Table 1:** U-values for the various construction parts based on the status quo and the new construction scenario.

Construction Part	Status quo U-value [W/m^2^K]	New construction U-value [W/m^2^K]
Exterior wall (EW)	1.50	0.15
Base plate (BP)	1.20	0.15
Flat roof (FRO)	1.00	0.15
Window (WIN)	2.90	1.20

**Fig. 2:**
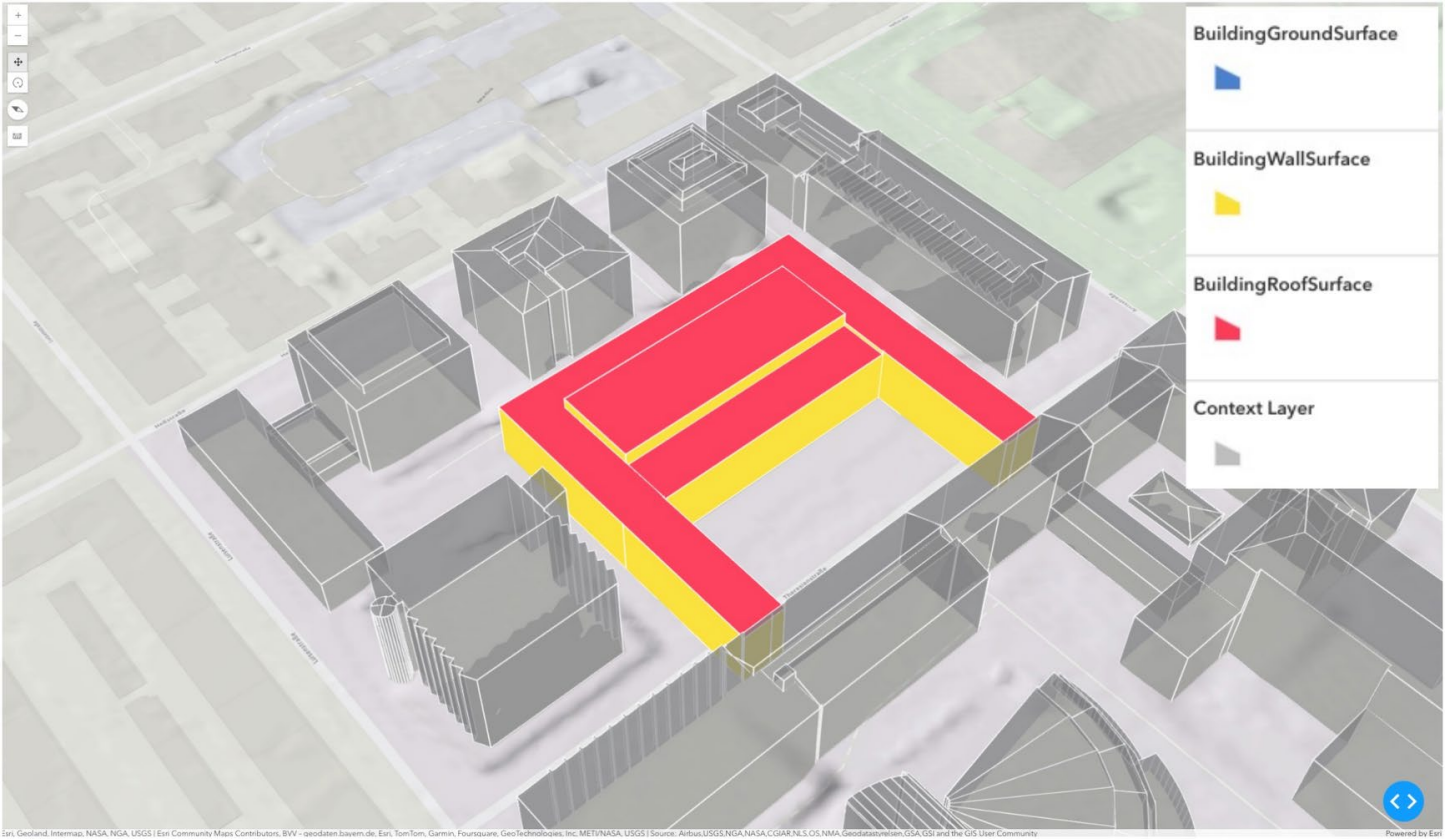
Illustration of the investigated building and the various construction parts.

A deep learning-based surrogate modeling approach was employed to evaluate the projected energy performance following the new construction in the case study. As a comparison, the energy performance of the status quo case is evaluated with the same approach. The energy model was trained on building energy simulation data generated through EnergyPlus for representative zones, as described in the authors’ previous work^47^. The geometry of these zones was derived using open data in the CityGML Level of Detail 2 (LoD2) format. From this data, the surface areas for the roof, ground, base, and shared wall surfaces were calculated, see Fig. 2. Specifically, the case study building has a BP of 4699 m$^2$, FL of 15611 m$^2$, EW of 3865 m$^2$, FRO of 4699 m$^2$ and WIN of 3104 m$^2$. The window area was estimated based on assumptions from^45^. Based on the geometry and number of stories, the building was divided into distinct zones. Each zone identified from the LoD2 object was assigned four usage profiles: laboratories, group offices, lecture halls, and circulation areas based on own assumptions regarding the proportions of each type of usage and the research in EnOB:dataNWG^45^. The occupant behavior, heating schedules and the internal heat gains were then modeled according to the four representative profiles specified in DIN V 18599-10^48^. Subsequently, the net energy demand from the surrogate model is first converted to end-use energy using annual efficiency estimates from^49^, then to primary energy demand according to specific heating options per DIN V 18599-10^48^. The building originally relies on district heating from natural gas-powered heat and power co-generation (CHP). In the new construction, alternative heating options are evaluated.

### DLCIA calculation

In the current LCA practices in the building industry, the GWP, expressed in kg CO$_2$-eq., is commonly used to indicate the environmental impact of different greenhouse gases relative to a reference gas, CO$_2$, through the state-of-the-art life cycle impact assessment (LCIA) calculation. In general, GWP of each gas $i$ is derived from its AGWP, as in Eq. (1). In this study, we use the AGWP based on the IPCC AR 6^50^ to perform the DLCIA calculation.

1 $$\begin{aligned} \begin{aligned} \textit{GWP}_i(H) = \dfrac{{\textit{AGWP}_i(H)}}{\textit{AGWP}_{CO_2}} \\ \end{aligned} \end{aligned}$$

where AGWP represents the integrated radiative forcing of each gas over time and is given in W m$^{-2}$ year. It can be derived through Eq. (2):

2 $$\begin{aligned} \begin{aligned}&\textit{AGWP}_i(H) = \int _{0}^{H} \textit{RF}_i(t) \,dt = \int _{0}^{H} \textit{RE}_{i}R_{i}(t) \,dt\\&\text {with} \quad R_{i}(t) = \exp (-\dfrac{t}{\tau _{i}}) \end{aligned} \end{aligned}$$

where $RE_{i}$ is the constant radiative efficiency (RE), indicating the unit radiative forcing of each gas. $R_{i}(t)$ represents the emission impulse remaining in the atmosphere over time and is expressed based on an exponential decay. Here, $\tau _i$ is the perturbation time scale of each gas.

In addition, the carbon cycle feedback is included in the calculation of AGWP and modeled differently for CO$_2$ and non-CO$_2$ gases. As in IPCC AR 5 and AR 6^43,50^, the carbon cycle feedback of CO$_2$ is modeled through a fitted $R_{i}$, with the fitting parameters $\tau _{i,n}$ and $\alpha _{i,n}$ (Eq. 3), adopted from Joos et al.^51^.

3 $$\begin{aligned} \begin{aligned} R_{i}(t) = \alpha _{i,0}+\sum _{n=1}^{N}\alpha _{i,n \cdot }\exp (-\dfrac{t}{\tau _{i,n}}) \end{aligned} \end{aligned}$$

As for the non-CO$_2$ gases, the carbon cycle feedback is modeled through a nested convolution of climate-carbon feedback IRF $\gamma R_{F}$, AGWP and absolute global temperature potential (AGTP) (Eq. 4). The climate-carbon feedback instantaneous radiative forcing (IRF) $\gamma R_{F}$ is calculated through Eq. (5). The methodology and parametrization are based on Gasser et al.^52^. Subsequently, the $\Delta AGWP$ is treated as an additional value to be aggregated to the AGWP derived from Eq. (2).

4 $$\begin{aligned} & \begin{aligned} \Delta \textit{AGWP}_i(H) = \int _{0}^{H} \gamma R_{F}(H-t) \int _{0}^{t} \textit{AGTP}_{i}(\tau ) \cdot \textit{AGWP}_{CO_2}(t-\tau ) \, d\tau \, dt \end{aligned} \end{aligned}$$

5 $$\begin{aligned} & \begin{aligned} \gamma R_{F}(t) = \gamma \delta (t) - \gamma (\dfrac{\alpha _1}{\tau _1}\exp (-\dfrac{t}{\tau 1})+\dfrac{\alpha _2}{\tau _2}\exp (-\dfrac{t}{\tau 2})+\dfrac{\alpha _3}{\tau _3}\exp (-\dfrac{t}{\tau 3})) \end{aligned} \end{aligned}$$

Here, AGTP is calculated through a convolution integral of the aforementioned IRF $R_{i}$ of each gas. It is important to mention that $\delta (t)$ from Eq. (5) is a Dirac-$\delta$ function to approximate the CO$_2$ flux at $t=$0. To practically model it and to reduce the computational cost in the calculation, we perform the calculation numerically, with a time stamp of 0.1 year. Accordingly, the Dirac-$\delta$ function $\delta (t)$ is approximated as 1 divided by the time stamp. The calculation method is applied to CO$_2$, CH$_4$, N$_2$O and 245 halogen gases, aligned with IPCC AR 6^50^.

**Fig. 3:**
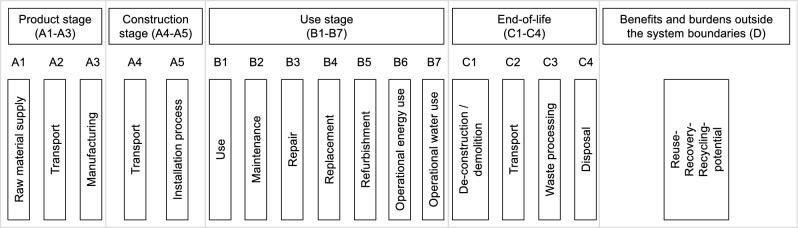
Life cycle phases of a building.

By using the dynamic AGWP obtained through the above method, the DLCIA calculation for the building is performed. Similar to that in the static LCA approach, different life cycle phases are considered according to EN 15978^53^, as shown in Fig. 3. The investigated life cycle phases in this study include product (A1–A3), replacement (B4), operational energy use (B6) and EoL (C). Accordingly, the basic calculation method can be expressed using Eq. (6):

6 $$\begin{aligned} \begin{aligned} EI = \sum _{j=1}^{N}(m_{j,a}\, \, m_{j,b} \,\, m_{j,c} ) \cdot \begin{pmatrix} \textit{GWP}_{j,a}\\ \textit{GWP}_{j,b}\\ \textit{GWP}_{j,c}\\ \end{pmatrix} \end{aligned} \end{aligned}$$

where $EI$ is the overall environmental impact of $N$ investigated materials. $m_{j,x}$ and ${GWP}_{j,x}$ stand for the material mass and unit environmental impact aggregated for a certain period of time, e.g., 100 years as in $GWP_{100}$, of each life cycle phase of each material. Herein, $a$, $b$ and $c$ represent the product, use and EoL phases of the life cycle of each material, respectively. When applying the dynamic AGWP, Eq. (6) can be adopted and the material quantity $m_{j,x}$ is transformed to $M_{j,x}$ representing a set of various quantities subject to time. Accordingly, the calculation of each material’s embedded environmental impact is shown in Eq. (7):

7 $$\begin{aligned} \begin{aligned}&EI_j = (M_{j,a}\, \, M_{j,b4} \, \, M_{j,c} ) \cdot \begin{pmatrix} \textit{AGWP}_{j,a}(RSP)\\ \textit{AGWP}_{j,b4}(RSP)\\ \textit{AGWP}_{j,c}(RSP)\\ \end{pmatrix}\\&\text {with}\\&M_{j,a} = (m_{j,a}^{t=0 \cdot RSL}, \, ..., m_{j,a}^{t=\epsilon \cdot RSL})\\&M_{j,b4} = (m_{j,b4}^{t=0 \cdot RSL}, \, ..., m_{j,b4}^{t=\epsilon \cdot RSL})\\&M_{j,c} = (m_{j,c}^{t=1 \cdot RSL}, \, ..., m_{j,c}^{t=\epsilon \cdot RSL})\\&\epsilon = RSP/RSL \end{aligned} \end{aligned}$$

where RSP is the reference study period and RSL represents the required service life of each material, i.e., life span of each material. $\epsilon$ is defined by RSP and RSL and represents the replacement frequency of a material in the use phase. Subsequently, with the RSL and $\epsilon$ of each material, the exact time point $t$ for the material replacement can be defined. In this study, the RSP is 100 years, corresponding to the commonly applied $GWP_{100}$ representing the GWP over 100 years after impulse. As aforementioned, instead of a single value, the material mass is given as $M_{j,x}$ and the material quantity at specific time point is denoted as $m_{j,x}^{t}$. It is to note that in case of having a linear supply chain based on the principle of cradle to grave, the material mass of replacement in the use phase B4, $M_{j,b4}$, is identical as of the product phase $M_{j,a}$. As a result, the DLCIA result is derived based on the mass of a material subject to time, obtained through the predefined construction typologies, and the AGWP of the material, defined through a multiplication of AGWP of each gas and the material-specific LCI extracted from ecoinvent.

As for the environmental impact during operation, ${EI}_{operation}$, $m_{b6}$ represents the annual primary energy demand as the life cycle stage B6 and the calculation is expressed by Eq. (8).

8 $$\begin{aligned} \begin{aligned} {EI}_{operation} = M_{b6} \cdot {AGWP}_{b6} (RSP) \,\,\,\text {with} \,\,\,M_{b6} = (m_{b6}^{t=0}, \, ..., m_{b6}^{t=RSP})\\ \end{aligned} \end{aligned}$$

Using Eqs. (7) and (8) with both the cumulative and non-cumulative AGWP, we calculate both the integrated and temporal environmental impact of different materials, such that a comprehensive analysis for the building typology can be conducted.

### DLCIA analysis

After conducting the DLCIA calculation, the environmental impact results can be shown and analyzed dynamically. In this study, both embedded and operational emissions are calculated and the results are analyzed on building, building component and building material levels, respectively. To gain a deep insight in the material characteristics, the analyses include the AGWP value, which shows the total environmental impact, along with the slope of non-cumulative AGWP over time and the dynamic share of different building components or materials that present the trend of emission impulse. Based on these analysis criteria that are either examined individually or in combination, the material characteristics can be further understood and improvement solutions can be potentially guided.

### Modeling of improvement solutions

**Fig. 4:**
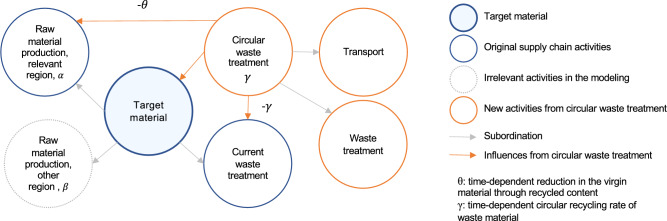
Modeling principle of the circular recycling.

After the DLCIA analysis, different improvement solutions are modeled and evaluated. In this study, the improvement solutions primarily focus on the building materials. Specifically, the improvement solutions focus on the product and EoL phases, which also directly influences the results of material replacements by managing the waste from the replaced materials and integrating new materials. Improvement solutions in the product phase include: (1) reduction of material quantities, e.g., through lightweight construction with hollow structure; (2) use of alternative materials, e.g., alternative insulation materials. It is important to note that this study does not consider the impact of material changes on the U-values of the building envelope. The consequent influence on operational energy use was excluded to avoid conflating dynamic effects and to maintain a clear focus on the DLCIA of embodied emissions. As for the EoL phase, the improvement solutions are based on the future estimation of a higher circular recycling rate responding to the promoted concept of circular economy. In the current modeling strategy that mirrors the current recycling situation, deconstructed materials after their RSLs are usually fed into another product supply chain. Accordingly, the EoL and product phases of one material for replacement are usually separately calculated. This modeling strategy often results in a simple cut-off in the product phase and the neglect of the burdens occurring during the waste treatment when considering recycling measures. To address this, we model a circular recycling process that reintegrates the treated demolition waste after the material replacement into the production of virgin materials for the same product. For material replacement after the initial material reach its RSL, we assume that the replacement materials are also produced using the circularly recycled materials, following the same recycling strategy. Accordingly, the evaluation of one material not only considers the further treatment of demolition waste in the EoL phase to prepare for the circular recycling, but it also reduces the demand for virgin materials to produce the replacement material. As the waste treatment and replacement happen in the future, we identify the recycling rate for the relevant years dynamically to more accurately model this process. Specifically, an annual increase of the reduction in the virgin material of 1% is estimated based on the material reduction goal from the Swiss federal Circular Gap Report^54^. Based on this and by also considering the subtraction of waste materials throughout the supply chain that can be directly reused in the product phase without further treatment, an annual increase in the circular recycling rate is defined accordingly. Subsequently, the circular recycling rate and reduction rate in the virgin material at each replacement point are defined. Figure 4 shows the modeling principle of the circular recycling with the influences on the initial waste treatment process and the production process with virgin materials. Here, $\gamma$ stands for the circular recycling rate and $\theta$ represents the reduction in the virgin material subject to time.

The influences of the improvement solutions can be calculated using equation (7). For the improvement solutions in the product phase, the initial $M_{j,a}$ and $M_{j,b4}$ are changed by the material quantity reduction. The initial $AGWP_{j,a}$ and $AGWP_{j,b4}$ are replaced by alternative AGWP due to the use of alternative materials. Herein, the individual material mass $m_{j,a}^{t}$ and $m_{j,b4}^{t}$ remains identical due to the linear supply chain. As for the improvement solutions in the EoL phase, Eq. (7) is used for each raw material production or relevant component of each investigated target material. Specifically, the mass of the raw material production or relevant component at each replacement point, $m_{j,b4}^{t}$, is not identical as $m_{j,a}^{t}$ and is gradually reduced by the reduction rate $\theta$ of the circular recycling at each replacement point. Here, this reduction in the raw material is limited by its maximum portion in the investigated material, i.e., the reduction rate $\theta$ at any time point should not exceed the original portion of the raw material, $\alpha$. Accordingly, the circular recycling rate and the reduction rate remain constant after $\theta$ reaches $\alpha$. The material mass in the EoL phase is modeled similarly. $m_{j,c}^{t}$ represents the quantity of either the initial waste treatment or the subsequent waste treatment necessary for the circular recycling, which are both determined by the circular recycling rate $\gamma$ at each replacement point (see Fig. 4). It is important to mention that the improvement solutions in the EoL phase do not influence the environmental impact at $t=0$, since the solutions are future driven.

### Cross-validation of GWP results: static versus dynamic calculation methodologies

**Fig. 5:**
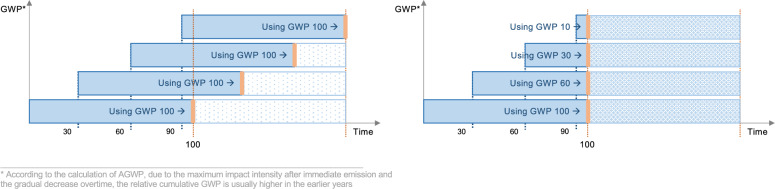
Schematic representation of GWP calculation using static and dynamic methodologies (left: static calculation, right: dynamic calculation).

To validate the DLCIA results, a comparative analysis was performed against the conventional static calculation. For better comparability, both methods were applied to calculate the GWP. Figure 5 shows a schematic representation of both methods applied to an example with a 30-year RSL. The conventional static approach uses a static characterization factor, i.e., $GWP_{100}$, applied to each material production and replacement event, regardless of its temporal occurrence within the RSP. This method aggregates impacts from discrete events as if they were simultaneous, neglecting the temporal sequence of emissions. As a result, it fails to account for the continuous decay of the GHGs in the atmosphere and their continuous impacts, treating the impact of an emission at time $t=0$ as identical to an emission at time $t=n$. In contrast, the dynamic GWP calculation uses time-dependent characterization factors derived from the AGWP via the DLCIA calculation. This approach calculates the relative GWP at the exact time of each emission event, accurately reflecting the changing radiative forcing effect of GHGs throughout the whole period. The dynamic calculation is detailed in Algorithm 1, where the DLCIA approach is represented by the function “CalculateDynamicImpact” and “Timeshift” models the material replacements. The time horizon (TH) varies according to the exact time point of the emission event, as shown in Fig. 5. In addition, for a direct comparison with the existing literature^38,40^, a second dynamic calculation is performed using the DLCIA results and a fixed 100-year TH characterization factor.

**Algorithm 1:**
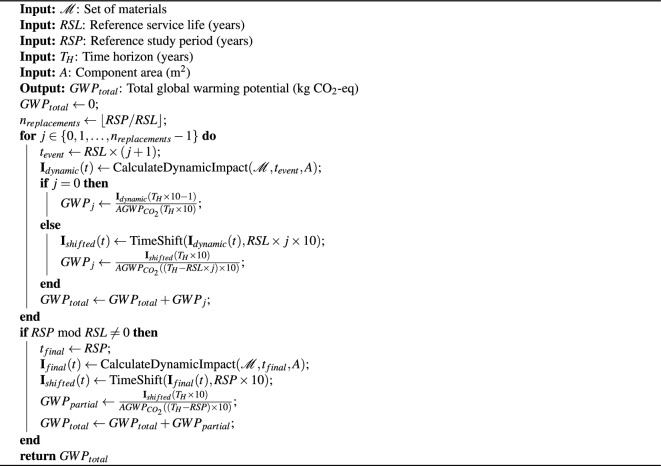
Algorithm of GWP calculation based on the AGWP results from the DLCIA calculation.

## Results

Following the afore-described methodology, the obtained results of the study include the following parts: (a) analysis results of the initial DLCIA calculation, (b) results after the improvement of building materials in the product phase, (c) results after the improvement of building materials in the EoL phase, (d) overall comparison of all improvement solutions, and e) comparative analysis of static and dynamic LCA results. In the following sections, the five parts of the results will be presented and discussed, respectively.

### DLCIA results and analysis

**Fig. 6:**
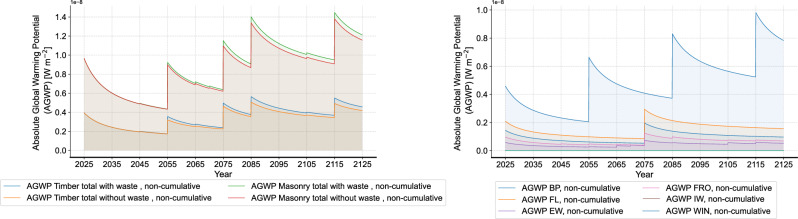
Results comparison on the building and building component level (left: overall comparison of timber and masonry construction, right: comparison of building components of masonry construction).

In the first step, the initial DLCIA results that show the total and dynamic AGWP are analyzed and additionally compared with an exemplary timber frame building typology (see Supplementary Material). Compared to the timber construction that has an AGWP over 100 years of $3.49 \times 10^{-7} \text{Wm}^{-2}\text{year}$, solid masonry construction exhibits a high total AGWP of $8.82 \times 10^{-7} \text{Wm}^{-2}\text{year}$. In addition, Fig. 6 presents the DLCIA results at the building and building component levels. The curves illustrate the change in the environmental impact, measured in AGWP, characterized by a sharp peak immediately following each emission event and a subsequent exponential decay. Each increase in the curve corresponds to a material replacement event, timed according to their respective RSLs. As shown, the dynamic non-cumulative AGWP of masonry construction is also higher over time. As on the building component level, it can be seen that the dynamic AGWP of the windows (WIN) strongly dominates, followed by floor/ceiling (FL), base plate (BP), flat roof (FRO) and exterior wall (EW).

**Fig. 7:**
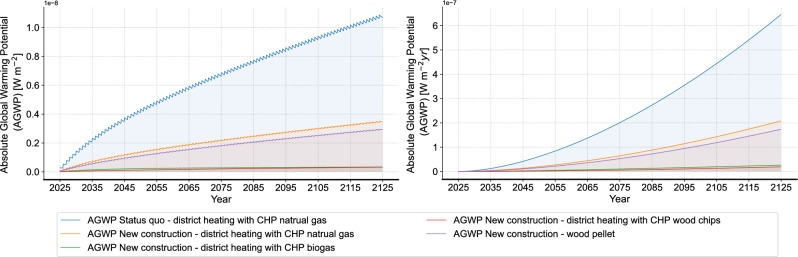
Results of environmental impacts during operation (left: non-cumulative, right: cumulative).

As for the operational phase, Fig. 7 shows the dynamic AGWP using the annual primary energy demand. Here, for the new construction in the case study, three energy supply options are evaluated: district heating with CHP driven by natural gas, biogas and wood chips, as well as furnace with wood pellet. In addition, the results are compared with the status quo case with the original building properties and natural gas-powered district heating with CHP. In general, different from the results of building materials, the continuous energy consumption makes the change cycle of the instantaneous AGWP shorter, which also leads to a more significant overall upward trend of the non-cumulative results. When comparing the four energy supply options, it can be observed that CHP with natural gas exhibits higher environmental impact than other heating options and the gap is increasing year by year. In addition, it is to note that despite the evident advantage of wood pellet furnace, CHP driven by biogas and wood chips show even lower environmental impacts. Thus, the environmental impact of district heating highly depends on the energy source, and similar sources can yield different results based on different supply systems.

**Fig. 8:**
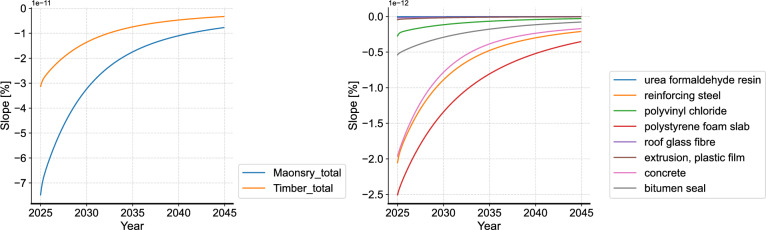
Analysis of curve slopes (left: comparison of timber and masonry construction, right: comparison of the materials in the flat roof of masonry construction (FROmas).

**Fig. 9:**
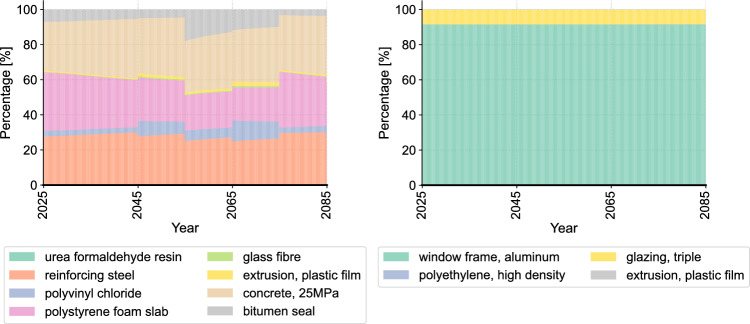
Analysis of time-dependent proportion (left: flat roof, right: aluminum window).

Next, we compare the curve slope of non-cumulative AGWP in different time periods to assess the trend of each material’s emission impulse (Fig. 8). Here, timber construction is taken again as a comparison for the building level analysis. It can be seen that despite the higher instantaneous AGWP, masonry construction shows a greater absolute slope and a more rapid decrease in the emission impulse overtime compared to timber construction. This finding shows that the environmental impact of masonry construction exceeds timber construction immediately after the initial impulse, yet drops faster. Considering the characteristics of exponential functions, it can be inferred that reducing material quantities is a potential improvement solution, in addition to directly switching to alternative construction materials. The sub-figure on the right of Fig. 8 shows the slope comparison of the materials in the flat roof. The result shows a higher initial value and a faster decrease in the emission impulse particularly in polystyrene foam slab, reinforcing steel and concrete. This different dynamic change of emission impulse of different materials can also be seen in the dynamic proportion in Fig. 9, where the proportion of polystyrene foam slab, reinforcing steel and concrete dynamically decreases, while the proportion of polyvinyl chloride and bitumen seal gradually increases. Accordingly, the reduction of the initial quantity of polystyrene foam slab, reinforcing steel and concrete can be an improvement solution for the flat roof to reduce the overall environmental impact. Different from the flat roof, the dynamic proportion of the different materials in the aluminum window does not show significant change over time. With the overall high proportion of aluminum window frame, alternative materials for the window frame can be considered as the improvement focus for this building component. Similarly, the analyses are conducted to all building components.

### Improvement solutions in the product phase

To reduce the environmental impact of the solid masonry building typology, improvement solutions in the different life cycle phases are modeled and evaluated. Based on the result analyses above, improvement solutions in the product phase focus on reinforced concrete, clay brick, polystyrene foam slab and aluminum window frame. To reduce the initial material quantities, we replace the reinforced concrete for the floor and roof slabs with hollow core concrete that leads to a material reduction of 30%^55^. For the clay brick in the exterior wall, we use hollow clay bricks with a material reduction of 20%^56^. And for the polystyrene foam, we choose the glass wool mat as an alternative material. Similarly, we use alu-wood window frame as an alternative material to replace the aluminum window frame. To better examine the effects, we test and analyze the improvement solutions separately and in combination. Table 2 shows an overview of the improvement solutions in the product phase.

**Table 2:** Overview of improvement solutions in the product phase.

No.	Improvement solutions in the product phase
1	Quantity reduction in reinforced concrete and clay brick
2	Glass wool mat as alternative material for polystyrene foam slab
3	Alu-wood window frame as alternative material for aluminum window frame

**Fig. 10:**
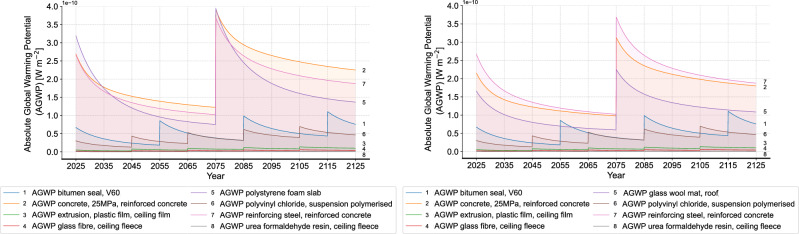
Results of materials in the flat roof (left: status quo, right: after improvement solutions in the product phase).

**Fig. 11:**
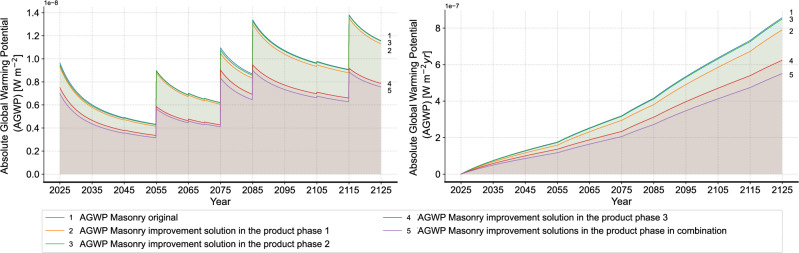
Result comparison of all improvement solutions in the product phase (left: non-cumulative, right: cumulative).

Figure 10 shows the comparison of the dynamic non-cumulative AGWP results of materials in the flat roof before and after all the improvement solutions in the product phase. A noticeable reduction in the AGWP of concrete and reinforcing steel can be seen at both the start of RSP and the time of replacement. As for the insulation material, the replacement of polystyrene foam slab with glass wool mat also exhibits a reduction in AGWP at all relevant time points. Figure 11 presents an overall comparison of the total results for the entire building after implementing all improvement solutions in the product phase. As a result, all improvement solutions lead to a reduction in the AGWP of the masonry construction. Herein, improvement solution 2, by replacing the roof insulation of polystyrene foam slab with glass wool mat, results in the smallest AGWP reduction. This finding shows an insignificant influence of simply replacing the material and indicates the small difference between polystyrene foam slab and glass wool mat. However, this finding should be looked at carefully, since the polystyrene foam slab from ecoinvent already considers recycled content in the production, which can be different in practice depending on the different manufacturers. On the other hand, the biggest AGWP reduction can be seen in the improvement solution 3, which changes the aluminum window frame to alu-wood one. This great contribution of changing the window frame material also aligns with the high proportion of windows in the overall environmental impact, which can be explained by the high quantity of aluminum.

### Improvement solutions in the EoL phase

**Fig. 12:**
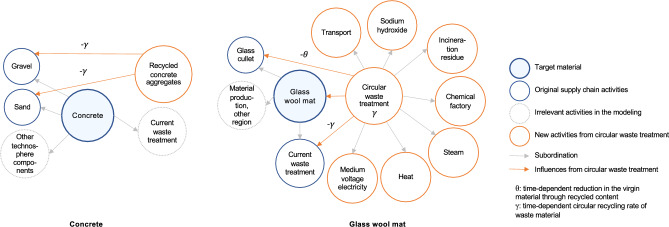
Modeling principle of the circular recycling of concrete and glass wool mat.

As aforemetioned, possible circular recycling measures are considered in the EoL phase. Based on the analysis results above and the practical applicability of circular recycling, we consider four materials for improvement in the EoL phase: glass wool mat, concrete, polyvinyl chloride film and triple glazing. The circular recycling considered in the study refers to processing collected waste materials after their RSLs are reached and reintegrating the processed waste materials into the next production cycle of the same product. As a result, the circularly recycled materials reduce certain amount of the virgin material that is originally needed in the product phase. This modeling approach directly influences the environmental impact results of the material replacements. Subsequently, with the respective annual increase in the circular recycling rate of each material (Table 3), the circular recycling processes and their influences are modeled. Figure 8 demonstrates the circular recycling of concrete and glass wool mat as an example. To circularly recycle the glass wool mat, pyrolysis with sodium hydroxide is applied, according to Wajima et al.^57^. After applying triple the mass of sodium hydroxide, 40% residual of soluble silicates can be obtained, which can potentially replace the glass cullet in the production phase of glass wool mat. Together with 9% replacement from spinning waste^58^ and cut-off materials from construction site, as well as the annual increase of the reduction in the virgin material of 1%, an annual increase in the circular recycling rate of 2.28% can be derived. Accordingly, the reduction in the virgin material $\theta$ and the circular recycling rate $\gamma$ can be determined subject to time. Different from glass wool mat, circular recycling of concrete considers the use of recycled gravel as concrete aggregates, where the further treatment is neglected, following the modeling principle of the activities considering recycled concrete aggregates in ecoinvent^59^. As a result, the reduction in the virgin material and the circular recycling rate are identical and are expressed by $\gamma$. As for the polyvinyl chloride film and triple glazing, re-molten polyvinyl chloride flake replaces the vinyl chloride and the collected glass cullet reduces the production of the uncoated flat glass (Fig. 12).

**Table 3:** Dynamic change in the recycling rate of the investigated materials.

Material	Pre-consumer waste	Usable part	Annual increase in the circular recycling rate	Maximum reduction in the targeted material ($\alpha$)
Glass wool mat	9% from spinning waste and cut-off	40 % residual ratio after pyrolysis	2.28 %	glass cullet: 1.14 %
Concrete	No pre-consumer waste considered	No material loss estimated	1 %	gravel: 51.65 %, sand: 29.13 %
Polyvinyl chloride film	No pre-consumer waste considered	No material loss estimated	1 %	vinyl chloride: 19.05 %, extrusion, plastic film: 100%
Triple glazing	30 % internal cullet in production	No material loss estimated	0.7 %	uncoated flat glass: 16 %

**Fig. 13:**
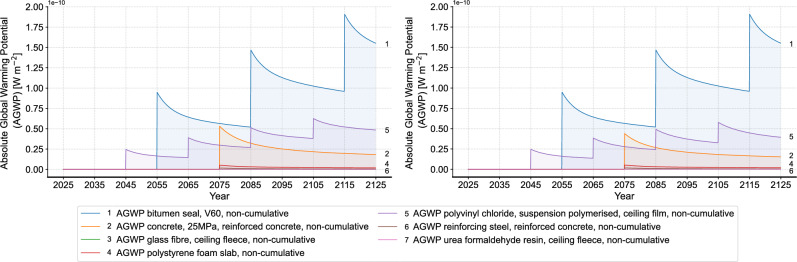
Comparison of environmental impact of waste treatment of flat roof before and after improvement solutions in the EoL phase (left: initial state, right: after improvement solutions in the EoL phase).

**Fig. 14:**
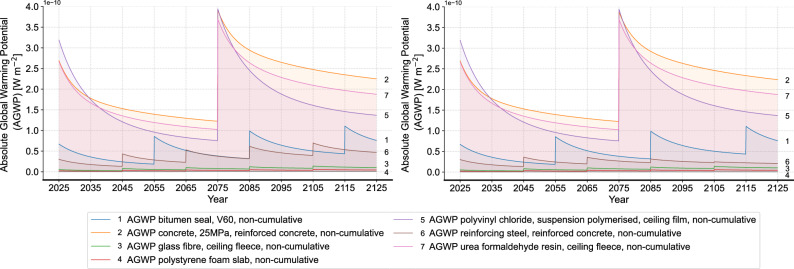
Comparison of environmental impact of production and replacement of flat roof without waste before and after improvement solutions in the EoL phase (left: initial state, right: after improvement solutions in the EoL phase).

Figures 13 and 14 present a comparison of the flat roof’s environmental impact before and after the improvement solutions in the EoL phase. Figure 13 shows the environmental impact of waste treatment in the EoL phase and Fig. 14 shows the results for production. It can be seen that the environmental impact of concrete slightly decreases after replacement due to the reduced input of gravel and sand, while the impact from the waste treatment remains unchanged. In contrast, the environmental impact of polyvinyl chloride film shows a year-by-year decline in both waste treatment and production after replacement, until the maximum reduction, i.e., the original portion in the material, is reached. The effectivity of circular recycling is thus more convincing through this dynamic illustration. The glass wool mat exhibits a distinct behavior in waste treatment and production after replacement (see Supplementary material). With pyrolysis as the further treatment, the environmental impact of circular recycling exceeds that of the initial waste treatment, while the replacement of the virgin material with the soluble silicates is minimal. This leads to a higher total environmental impact compared to the initial state, despite the circular recycling rate remaining unchanged after two years due to the low original portion of glass cullet. Therefore, in the following analyses, we divide the improvement solutions in the EoL phase into two scenarios: one with glass wool recycling and one without.

### Comparison of all improvement solutions

**Fig. 15:**
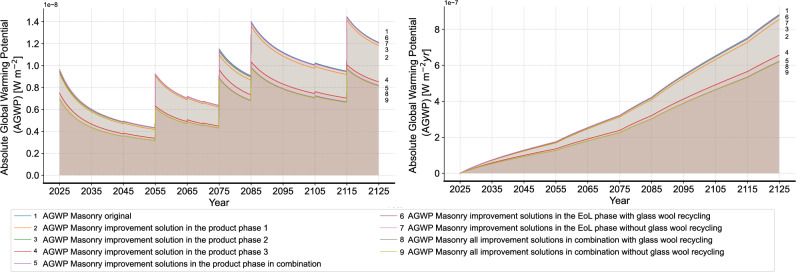
Results of all improvement solutions separately and in combination (left: non-cumulative, right: cumulative).

Figure 15 shows the results of all improvement solutions. In general, all improvement solutions exhibit a reduction in the environmental impact and the gap between the different improvement solutions gradually widens over time. Among all improvement solutions, the advantage of the improvement solution 3 in the product phase is the most evident. This finding highlights the importance of the window components of a building construction and reveals the irrationality of the customary binding of metal window frames and solid masonry construction. When examining the results of improvement solutions in the EoL phase, it can be observed that the overall environmental impact is lower when excluding the circular recycling of glass wool mat compared to the scenario with glass wool recycling. As previously noted, the current limitations in glass wool recycling technology result in higher resource input and a limited recovery rate, ultimately leading to a greater environmental impact. However, the difference between the two scenarios, with and without glass wool recycling, is minimal. This is due to the unchanged circular recycling rate of glass wool mat after two years because of the limited maximum reduction in glass cullet, thereby preventing ineffective circular recycling.

**Fig. 16:**
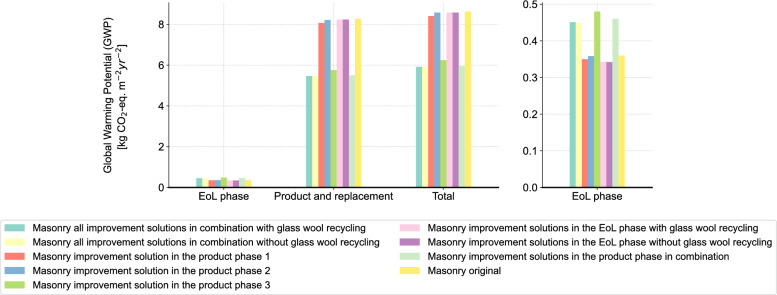
GWP results of all improvement solutions in different phases.

To further understand the results, we compare the performance of the different solutions in both the product and EoL phases. Figure 16 summarizes the environmental impact results over the whole RSP in the form of GWP per square meter use area and year, zoomed into the respective phases. It is to notice that the dynamic calculation using the dynamic characterization factor derived from the AGWP results is applied, as described in Section 2.5. A significant difference between the product and EoL phases is evident in all cases. This difference is primarily due to the industry’s reliance on “simple” waste treatments, such as landfill and incineration. This imbalance highlights the higher burden during the product phase and explains the larger benefits of reducing initial material quantities in the product phase. In addition, it is to observe that the environmental impacts in the EoL phase are also diminished after the improvement solutions 1 and 2, due to the reduced material quantity. This further underscores the significant advantages of these improvement solutions. Specifically in improvement solution 3 in the product phase, using alu-wood window frame results in a higher GWP in the EoL due to the higher mass compared to aluminum window frame for the same area. Nevertheless, this is offset by a 30.35% reduction in GWP during the product phase, which mitigates this relatively minor drawback. As for the results of the improvement solutions in the EoL phase, an insignificant reduction in the environmental impact in the product phase and a higher or unchanged impact in the EoL phase can be observed. These outcomes reveal the low conversion rate from recycled waste to the virgin material and explain the limited willingness of implementing circular recycling in practice. To address this, more efforts in recycling technology are necessary to improve the conversion rate of recycling and reduce the difference between the product and EoL phases. In total, combining all improvement solutions without the circular recycling of glass wool mat results in a total GWP of 5.91 kg $\text{CO}_2$-eq. $\text{m}^{-2} \text{year}^{-2}$ and a reduction of 31.52% compared to the initial state. As a result, the gap between the solid masonry construction and timber frame construction is reduced.

### Comparative analysis of static and dynamic LCA results

To validate the results, the GWPs derived from the dynamic and static LCA calculations were compared (Table 4). The dynamic GWP results, which applies the time-dependent characterization factor, were consistently higher than those from the static calculation. For solid masonry construction, the dynamic results were approximately 5% higher, while a higher difference of about 7% was observed for timber frame construction. The difference between the static and dynamic calculation results arises because the AGWP is highest immediately following an emission and decays over time. Consequently, emissions occurring earlier in the life cycle show a stronger influence on the relative cumulative radiative forcing. The dynamic method captures this temporal effect by applying time-dependent characterization factors, and provides a more accurate assessment of the environmental impact of emissions at different points in time. Furthermore, a third scenario was evaluated using a dynamic calculation with DLCIA results and a fixed 100-year TH characterization factor. The results from this approach were generally lower than both the static and time-dependent dynamic calculations. This finding aligns with previous studies^38,40^ and can be explained by the truncation of impacts that occur after the 100-year threshold, failing to account for the full duration of GHG effects.

**Table 4:** Comparison of GWP results from static and dynamic calculation.

Calculation	Timber status quo (kg CO_2_-eq. m$^{-2}$year$^{-2}$)	Masonry status quo (kg CO_2_-eq. m$^{-2}$year$^{-2}$)	Masonry after improvements (kg CO_2_-eq. m$^{-2}$year$^{-2}$)
Static calculation	3.16	8.23	5.65
Dynamic calculation with time dependent characterization factor	3.38	8.63	5.91
Dynamic calculation with a TH of 100 years	2.18	5.52	3.87

## Discussion

In this study, we applied a DLCIA approach to investigate the environmental impact of a case study building with different improvement solutions. While most existing studies either applied presumed mathematical functions or the calculation methodology from IPCC Assessment Report 5^43^ or earlier versions that does not consider the carbon response of other gases besides CO$_2$, we apply the calculation methodology according to the newest IPCC AR 6, including the carbon response of all GHG gases. Different from the conventional calculation method using a cumulative value, e.g., $GWP_{100}$, given in kg CO$_2$-eq., the presented study uses the AGWP, given in Wm$^{-2}$ year, to reveal the original calculation logic of GWP and enhances the understanding of the concept. The results were further validated through a comparative analysis of the GWP results based on the conventional static calculation using $GWP_{100}$ and the dynamic characterization factors derived from AGWP results of the DLCIA approach. The results show that the GWPs using the time-dependent characterization factor based on the DLCIA approach are consistently 5–7% higher than those from the static calculation. This finding proves that the dynamic calculation of GWP based on the DLCIA approach can provide more accurate results of the environmental impacts in terms of capture the environmental impact at the exact time point of the emission events. The results of the dynamic calculation with a TH of 100 years resonate with the findings from the previous studies^38,40^, showing that applying the characterization factor according to the TH applied in the DLCIA tends to underestimate the impact. While Zieger et al. and Cordier et al. suggested performing the DLCIA for a longer TH and apply the characterization factors of a TH of 500 years to calculate the GWP, we recommend using the DLCIA approach to calculate the time-dependent characterization factor, which can correctly and effectively profit from the advantage of the DLCIA approach. Compared to prior research, this study also emphasizes the practical application of DLCIA in the building industry, guiding the development of effective solutions to mitigate a building’s environmental impact. The environmental impact throughout the whole life cycle can be better demonstrated and the DLCIA results can serve as a more convincing tool to explain the improvement solutions. In addition, different from the current LCA approaches that usually calculate product and EoL phases separately, without considering the relationships between the phases, we integrate the dynamic circular recycling in the study to model the supply chain. Unlike the previous study^27^ that uses system dynamics modeling to model the dynamic influence of circular recycling measures only focusing on each material, this study models the dynamic change considering the building context and replacement frequency, which demonstrates a more realistic life cycle. In total, DLCIA helps to gain a better understanding of the LCA results and the decision-makers can be better informed to achieve a more sustainable construction industry.

Besides the DLCIA approach, we gain some invaluable findings from the improvement results. With the combination of the improvement solutions, without the circular recycling of glass wool mat, a reduction of 31.52% in the total GWP can be achieved. This result proves the effectivity of reducing the environmental impact of the solid masonry construction without completely switching to alternative construction typologies. The significant contribution of replacing the aluminum window frame to alu-wood one reveals the high impact of aluminum and partially explained the high environmental impact of solid masonry construction due to the customary binding of metal window frames and solid masonry construction. Therefore, we suggest to separately evaluate window construction when comparing the environmental impact of different building construction typologies. Second, the reduction of material quantity through light weight construction is a potential direction in developing a sustainable masonry construction, as it affects both product and EoL phases in the whole life cycle. Thus, it is important to use statical optimization approaches to change the current component structure with solid materials^60^. As for the circular recycling measures, two findings are derived from the study. First, the actual reduction potential in the virgin material highly influences the potential of circular recycling. Specifically, the significance of circular recycling decreases as the growth of tangible benefits stops, which should be considered in modeling the concept of circular economy. As an example of this study, the dynamic calculation of circular recycling rate effectively allows a “stop-pure-loss” point, where the increment of circular recycling rate stops. As a result, the concept of circular economy can be more realistically presented and the decision-making towards circular economy can be more plausibly supported. Despite this modeling, the advantage of circular recycling is still minor, which leads to the second finding. We find out that the conversion rate of producing virgin materials using the recycled waste is relatively low following the current state of the art technologies. The merely slightly lower GWP results compared to the initial state prove that the low willingness in the market of practically recycling certain materials is not only due to the low economic benefit. To change the situation, it is important to increase the conversion rate of recycling measures and reduce the difference between the EoL and product phases. Through the dynamic modeling strategy from the study, it is possible to further examine the effectivity of the recycling measures in the future study.

The current study also has some limitations and certain aspects can be further deepened in the future study. In this study, we used building component assemblies based on the German architecture and the generic activities from ecoinvent to perform the DLCIA and model the improvement solutions. Therefore, we encourage other researchers to use activities with project-specific data to further validate the findings from this study. Second, we only considered material replacement after the RSL, without considering the retrofitting strategies. In future studies, it is important to integrate the retrofitting strategies with the time-dependent standards in the timeline. In addition, despite the consideration of both embedded and operational emissions, the focus of the improvement solutions lies in the building construction. Thus, it is of interest to use the presented methodology to investigate the interactions between building construction and HVAC systems^61,62^. Particularly, the interaction between the building construction and HVAC systems should be coupled with future retrofit measures. This integration can be realized through a feedback loop, where updated U-values resulting from insulation retrofits are used as inputs to the deep learning-based surrogate model employed in this study. As for the circular recycling measures, some assumptions made need to be validated with more accurate practical data from the industry. For example, the real pyrolysis processes or similar recycling processes of glass wool need to be examined and adjusted. New pioneering companies are recently founded to investigate measures to convert waste glass wool into glass cullet^63^; new studies are discussing the possibility of using water-based solvolysis to directly clean both the binders and glass fibers in the glass wool^64^. Also, while some materials can be recycled multiple times without clear signs of degradation, such as polyvinyl chloride films^65^, it is important to further investigate the degradation risk of recycling certain materials, such as glass product. In this context, more studies and practical data are required to more accurately model the recycling processes using the proposed methodology in the study. Furthermore, the proposed modeling strategy of circular recycling should be applied in a broader context to allow the modeling of material flow among multiple building entities to mitigate the limited reduction potential in each building material.

In general, despite primarily considering only one background dynamic factor, the dynamic environmental impact, this study uses DLCIA approach to reveal the importance of a time-dependent demonstration of environmental impact to guide decisions. In addition, the integration of the dynamic circular recycling in the DLCIA approach shows the potential of combining different dynamic factors and effectively presenting the outcome. In future studies of DLCA, DLCIA should serve as a basis, and other dynamic factors, such as the dynamic circular recycling strategy in this study, should be incorporated into the modeling to systematically perform DLCA for buildings.

## Conclusion

To enhance the plausibility of the current LCA for buildings, many studies were conducted to develop possible DLCA methodologies. In this study, we focus on one background dynamic factor, dynamic environmental impact calculation, i.e., DLCIA with a transparent and clear representation and comparison with the conventional static LCA approaches to highlight the importance of DLCIA in building planning. We apply it to evaluate a case study building with solid masonry construction with different improvement solutions in both the product and EoL phases of building materials, which also directly influence the replacement phase. The results demonstrate that DLCIA helps to better understand the material characteristics and can guide improvement solutions to reduce environmental impacts. In addition, the improvement solutions can be better demonstrated to support decision-making. Specifically, we prove that DLCIA provided a more accurate temporal representation of emission impacts, showing that dynamic GWP calculations are consistently 5% to 7% higher than their static counterparts. Based on the DLCIA results and analyses, the developed improvement solutions are able to achieve a 31.52% reduction in the total GWP. In future DLCA studies, DLCIA should be integrated with other dynamic factors to enable more systematic calculations. Furthermore, by integrating the dynamic circular recycling strategy in the EoL phase, the potential of combining different dynamic factors is highlighted and the concept of circular economy is critically discussed. To effectively promote the circular economy, it is essential not only to increase the conversion rate from waste materials to compounds for virgin materials but also to apply the proposed circular recycling modeling in a broader context to explore cross-material recycling and recycling among multiple building entities within the construction industry. This will help mitigate the limited reduction potential of individual building materials though circularly recycled waste materials. As a result, a more sustainable construction life cycle can be achieved.

## Data Availability

The datasets used and analyzed during the current study are available from the corresponding author on reasonable request.

## Abbreviation

AGTP: Absolute global temperature potential
AGWP: Absolute global warming potential
BP: Base plate
CHP: Heat and power co-generation
DIN: German Institute for Standardization
DLCA: Life cycle assessment
DLCIA: Life cycle impact assessment
EI: Environmental impact
EoL: End-of-life
EW: Exterior wall
FL: Floor
FRO: Flat roof
GHG: Greenhouse gas
GWP: Global warming potential
HVAC: Heating, ventilation and air conditioning
IPCC AR: Climate change assessment report
IRF: Instantaneous radiative forcing
LCA: Life cycle assessment
LCIA: Life cycle impact assessment
LoD2: Level of detail 2
RE: Radiative efficiency
RSL: Reference service life
RSP: Reference study period
TH: Time horizon
WIN: Window
